# United Nations meeting on antimicrobial resistance

**DOI:** 10.2471/BLT.16.020916

**Published:** 2016-09-01

## Abstract

World leaders gather at the United Nations General Assembly in New York this month to mount a response to the growing threat of antimicrobial resistance. Gary Humphreys and Fiona Fleck report.

Dr Abdul Ghafur, a consultant in infectious diseases in Chennai, had become alarmed by the number of multidrug–resistant infections in patients at major hospitals across India. 

In desperation, he and his colleagues issued the Chennai Declaration in 2012, calling on policy-makers to take action to address the growing problem in India. 

Four years later little has changed and now they fear that patients in India may be acquiring infections that are resistant to an antibiotic of last resort, colistin. 

Due to its high toxicity, colistin has been used primarily as a growth promoter for livestock. However, as more bacteria become resistant to other classes of antibiotics, colistin is increasingly being used in humans. 

The widespread use of colistin in animal farming has resulted in resistant infections that are spreading fast and weakening its power as a drug of last resort. 

“Colistin is the only treatment option with a reliable effect against multidrug-resistant bacteria in many regions,” says Professor Otto Cars of Uppsala University, Sweden, the founding director of ReAct, an independent network campaigning for global action on antibiotic resistance. “Losing it would put many lives at risk.”

Ever since antibiotics were developed in the 1940s, scientists have warned that using them improperly leads to bacterial resistance and that overuse of antibiotics only increases that risk because microbes naturally adapt to their environment. 

Today’s threat of widespread antimicrobial resistance (AMR) – in bacteria, parasites and viruses that cause infections and disease – raises the prospect of a world without effective antimicrobials, where a patient can die from previously treatable infections. 

This month global leaders gather at the United Nations General Assembly in New York to discuss the AMR problem and agree on a response.

For Dr Marc Sprenger, director of the World Health Organization’s AMR Secretariat, the United Nations gathering on 21 September represents a significant ramping up of the global response.

“There has been discussion of AMR in WHO since the 1960s, and plans since 2000, but it is now shifting from being a technical problem to a much higher level political issue,” Sprenger says, adding that this shift and the broad consensus on what needs to be done are reasons for optimism. 

The gathering in New York is the result of efforts led by WHO, the Food and Agriculture Organization (FAO), the World Organisation for Animal Health and several governments. It comes in the wake of national, regional and international initiatives, including the Chennai Declaration, regional declarations, World Health Assembly resolutions and gatherings of high-ranking decision-makers across the world. 

Recent high-profile reports warn of the dangers of not taking action. A bleak report by economist Jim O’Neill, commissioned by the British government and released in May, estimates that 700 000 deaths globally could be attributed to AMR this year and that the annual toll would climb to 10 million deaths in the next 35 years. The report projects US$ 100 trillion in losses by 2050 if nothing is done to reverse the trend.

WHO’s *Global action plan on antimicrobial resistance*, adopted at the World Health Assembly last year by Member States, proposes a way forward.

The plan has five objectives: to improve awareness and understanding of antimicrobial resistance in policy-makers and professionals; to strengthen surveillance and research; to reduce infection; to encourage the rational use of medicines in human and animal health care; and to increase investment in developing new medicines, diagnostics and vaccines.

Key to making the plan work is the “one-health” approach that reflects the links between human health, animal health and the environment, and requires many different sectors to come together to address the problem.

Under the plan, WHO Member States have until May 2017 to adopt national AMR action plans. 

One of the toughest challenges is to reduce the use of antimicrobials as growth promoters in animal husbandry. 

Global consumption of antimicrobials in food animals was estimated at 63 151 tons in 2010, of which the largest share, 23%, was in China, 13% in the United States of America, 9% in Brazil and 3% in India, according to Thomas Van Boekel and colleagues’ 2015 study in *Proceedings of the National Academy of Sciences of the United States of America*. 

The authors predict a 67% increase in the global consumption of antimicrobials by 2030, given the “shifting production practices in middle-income countries where extensive farming systems will be replaced by large-scale intensive farming operations”.

Use of antibiotics as animal growth promoters was banned in the European Union (EU) in 2006, although these are commonly used for mass prophylaxis in some countries. 

Even within the 28-country bloc there is a 100-fold variation in antibiotic use between countries, without apparent differences in the productivity of farms. Northern European countries, such as Denmark, the Netherlands, Norway and Sweden have made particular progress in developing food production systems using low levels of antibiotics. 

In June, EU member states adopted the one-health approach to antimicrobial resistance in a decision by the Council of the EU, calling on countries to comply with the ban on using antibiotics as growth promoters and to minimize their prophylactic use in farming.

“Countries with weak and poorly-resourced health systems will have the greatest problems in managing drug resistance.”

Marc Sprenger

“Across the world, countries are developing national AMR action plans,” Sprenger says, adding: “The lack of data for low- and middle-income countries means that we are unsure about the exact scale of the AMR problem, but that we have a problem is clear.”

“Most of the future burden of disease will be in Africa and Asia, and countries with weak and poorly-resourced health systems will have the greatest problems in managing drug resistance,” he says.

China is pushing forward on several fronts. Stand-out initiatives include the three-year campaign for rational antibiotic use launched by the National Health and Family Planning Commission of the People's Republic of China (NHFPC) in 2011.

China has also committed considerable resources to monitoring, having established more than 2400 monitoring network points that track the clinical application of antimicrobial drugs.

Surveillance from hospitals showed that the proportion of outpatients receiving prescriptions for antibiotics decreased from 22% to 14.7% between 2010 and 2012, and that of inpatients from 68.9% to 54%, according to Chinese research collaboration, the Collaborative Innovation Center for Diagnosis and Treatment of Infectious Diseases. The use of antibiotic prophylaxis in surgery decreased from 95% to 44.6% during the same time period.

China is also committed to the one-health approach to fighting AMR, with 12 ministerial departments meeting regularly to discuss progress, according to WHO’s China Country Office. These are led by the NHFPC and include Ministry of Agriculture representatives and others. 

According to the FAO in China, the government has issued several orders restricting the use of specific antibiotics in animal husbandry, but, despite this, tackling the misuse of antimicrobials in livestock is a challenge, as it affects the income of millions of households in rural areas. In poor areas, farmers tend to be more dependent on antimicrobials to control animal diseases and may struggle to reach a registered veterinarian and get a prescription on time. 

According to the FAO, a high-level veterinary committee discussed a study published in the *Lancet* in February by Yi-Yin Liu and colleagues linking the heavy use of colistin to promote livestock growth with the emergence of resistance to colistin in human bacterial infections. 

The committee agreed that a thorough risk analysis of AMR in the animal production system should be carried out. A 2016–2020 plan is expected to address the issue. 

China’s centralized government structure may also help the national policy response to AMR, but the challenge is implementation: making the policy a reality.

India released the *National policy for containment of AMR* in 2011 aiming to rationalize the use of antibiotics in hospitals, reduce over-the-counter sales of antibiotics and improve infection control in hospitals. 

But, while India’s national AMR policy was drawn up in New Delhi, individual states have considerable autonomy when it comes to implementation.

There have been some restrictions on the prescription of antibiotics for in-patients, but enforcement remains weak. According to Ghafur only a few of India’s 74 000 hospitals actually follow infection control guidelines.

“Some health centres report successful initiatives,” says Ghafur, “but there is a paucity of published data on whether Indian hospitals are compliant with existing policies.”

With an estimated 10–15% of *E. coli* infections and 30–40% of *K. pneumoniae* infections already resistant to standard treatment, India faces a new reality: that soon it may not be possible to treat these infections with colistin either.

“There has been a lot of denial about the gravity of the situation. Now it is time to act.”

Otto Cars

“That is the nightmare scenario,” says Cars, “and without concerted, global action, including perhaps legally binding conventions, it is a scenario we will have to face. There has been a lot of denial about the gravity of the situation. Now it is time to act.” 

**Figure:**
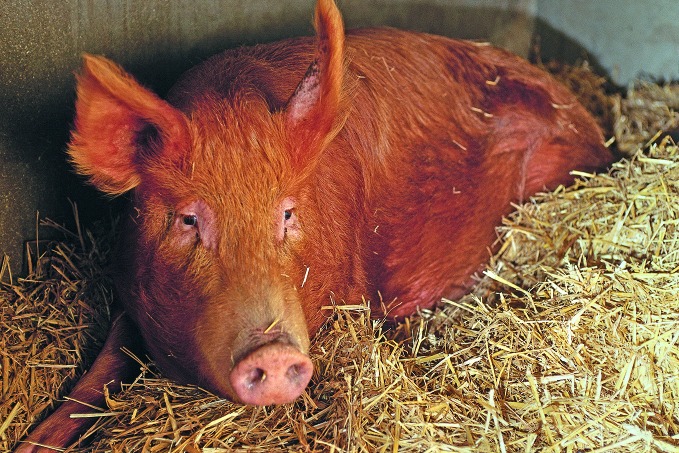
Pigsty at a farm in the United Kingdom. Pigsties have nearly become a thing of the past with the growth of intensive farming methods.

Wellcome Images/Anthea Sieveking

**Figure:**
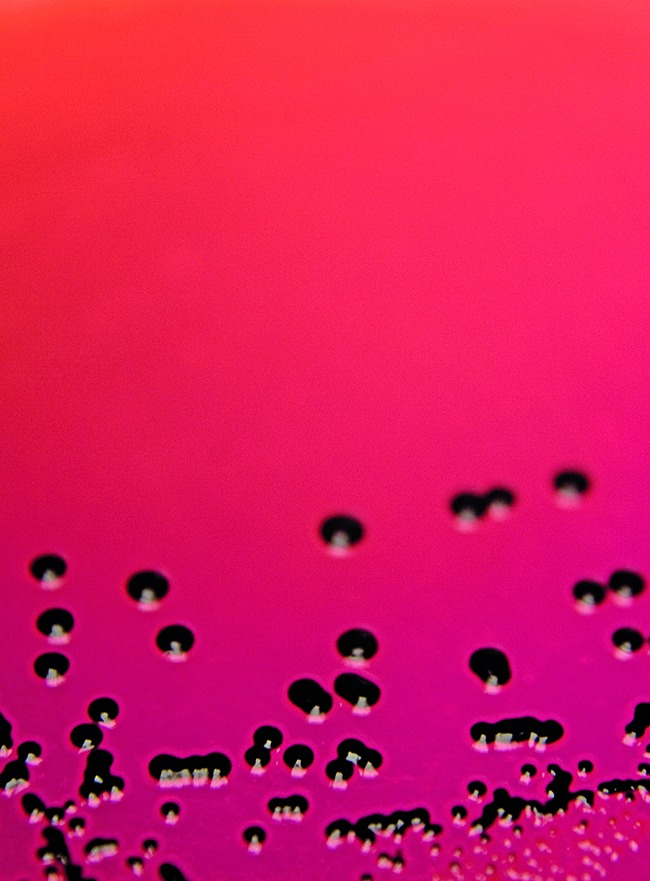
Colonies of *Salmonella typhimurium* DT104b, one of the pathogens with multiple antibiotic resistance genes.

Pablo Rojas

