# Corrigendum

**DOI:** 10.2471/BLT.16.101016

**Published:** 2016-10-01

In Volume 94, Issue 9, September 2016, page 639, the third paragraph should read: “Global consumption of antimicrobials in food animals was estimated at 63 151 tons in 2010, of which the largest share, 23%, was in China, 13% in the United States of America, 9% in Brazil and 3% in India, according to Thomas Van Boeckel and colleagues’ 2015 study in *Proceedings of the National Academy of Sciences of the United States of America*.”

